# Atlantic Horseshoe Crabs and Endotoxin Testing: Perspectives on Alternatives, sustainable Methods, and the 3Rs (Replacement, Reduction, and Refinement)

**DOI:** 10.3389/fmars.2020.582132

**Published:** 2020-09-30

**Authors:** Richard Gorman

**Affiliations:** Department of Geography, University of Exeter, Exeter, United Kingdom

**Keywords:** limulus amebocyte lysate, horseshoe crab (*Limulus polyphemus*), recombinant factor C, 3Rs (replacement, reduction, refinement), alternatives to animal testing, social science and humanities

## Abstract

Endotoxin testing is a vital part of quality and safety control in pharmaceutical production. The primary method for this testing in North America and Europe is the limulus amebocyte lysate (LAL) test, a critical component of which is the blood of Atlantic horseshoe crabs (*Limuius poiyphemus*). Procuring blood for LAL testing involves capturing and bleeding over 500,000 crabs from wild marine populations each year. Whilst efforts are made by manufacturers to return crabs to the sea following the collection of blood, there is a level of mortality and sub-lethal impact involved, prompting increasing discussions about welfare and ethics. The 3Rs – the ambition to where possible, replace, reduce, and refine the use of animals – are established and accepted worldwide as the best framework for governing animal-dependent science. However, the biomedical utilization of horseshoe crabs to produce the LAL test has rarely been viewed through a 3Rs framework. More recently, there has been a renewed attention on sustainable methods and alternatives to the LAL test. Drawing on in-depth qualitative interviews, this article examines stakeholder perspectives on opportunities for thinking with the 3Rs, considering current appetites to replace, refine, and reduce contemporary biomedical reliance on horseshoe crabs. The shape of conversations about the biomedical utilization of horseshoe crabs has shifted significantly in recent years, and the 3Rs are an important driver of change, offering the potential to advance the use of more sustainable methods, and realize the welfare considerations increasingly expected across science and society.

## Introduction

Atlantic horseshoe crabs (*Limulus polyphemus*) – though classed as ‘vulnerable’ (Smith et al., 2016) – play a vital role in enabling global public health. These blue-blooded marine invertebrates are, at least currently, used by humans to ensure that the vaccines, injectable medicines, and medical devices utilized in human and veterinary medicine are safe from contamination by endotoxins. Endotoxins, synonymously known as lipopolysaccharides (LPS), are molecules found as part of the outer membrane of Gram-negative bacteria, released upon the disruption of intact bacteria (through death or cell lysis). If released into the mammalian blood stream endotoxins are capable of producing a complex pattern of systemic toxicity, ranging from fever through to life threatening effects such as hypotension and shock (Levin et al., 2003). Endotoxins are highly heat-stable, and thus not destroyed as part of conventional sterilizing or heat-treating protocols. Indeed, sterilizing a product to kill any bacteria present can actually result in the release of LPS, and thus an essential – and regulatory mandated – part of quality and safety control in pharmaceutical production involves testing injectable drugs and medical devices for contamination by endotoxin (Joiner et al., 2002).

In North America and Europe the primary method for endotoxin testing is the limulus amebocyte lysate (LAL) test. ^1^This utilizes the coagulative properties of the blood of Atlantic horseshoe crabs to detect endotoxins, linking this immunologically unique and ancient species to the global supply chains of modern health and medicine. LAL is commonly understood and positioned as a ‘replacement’ itself, an alternative to the *in vivo* Rabbit Pyrogen Test (RPT), the previous regulatory standard for endotoxin testing, and thus development of the LAL test ostensibly led to a significant reduction in harm to animals (Hartung, 2001).

However, procuring blood for LAL testing involves capturing and bleeding over 500,000 crabs from wild populations each year, many of which die (Atlantic States Marine Fisheries Commission, 2019). The Atlantic States Marine Fishery Commission (ASMFC, the regulatory body around the horseshoe crab) assumes a 15% mortality rate for bled and released crabs. This 15% rate is highly debated and contested, ranging widely within published literature – Hurton and Berkson’s (2006) experiments resulted in a post-bleeding mortality rate of 8.3%, whilst Leschen and Correia’s (2010) study concluded a post-bleeding mortality rate as high as 29.8%.

The continued use – and potential rise in use given increasing global demand for pharmaceuticals – of horseshoe crabs is prompting growing questions around welfare and sustainability (Krisfalusi-Gannon et al., 2018). As alluded to earlier, the Atlantic horseshoe crab has been classed as ‘vulnerable’ by the IUCN, with populations trending to ‘decreasing’ (Smith et al. 2016), although the extent to which the biomedical use of crabs has an impact on population numbers is hotly debated and contested (Dawson and Hoffmeister, 2019). Whilst most crabs are returned to the sea following the collection of their blood, and some view the bleeding process as harmless, there are increasing discussions about the impact that capture and bleeding can have on crab health and mortality (Krisfalusi-Gannon et al., 2018; Maloney et al., 2018; Atlantic States Marine Fisheries Commission, 2019). Anderson et al. (2013) found that bled horseshoe crabs, post-release, suffered from significant changes in activity levels and behavioral rhythms, reductions in hemocyanin levels (potentially affecting immune function), and decreased reproductive fitness (prompting concerns about longer-term population impacts). Similarly, Owings et al. (2019) found that bled animals approached mating beaches less than control animals during the first week after release. Relatedly, there are growing debates around *Limulus’* sentience and capacity to suffer (John et al., 2018), with animal protection groups posing welfare and ethical questions about the continued use of horseshoe crabs within the pharmaceutical industry.

Despite the global reliance on this immunologically unique and ancient species and its centrality and indispensability amongst the supply chains of modern health and medicine, the pharmaceutical utilization ofhorseshoe crabs to produce the LAL test is rarely viewed through a 3Rs framework. The 3Rs – the ambition to where possible reduce, refine, and, replace the use of animals – are established and accepted worldwide as the best framework for governing animal-dependent science, playing an integral role in ensuring high standards of ethical consideration whilst also maximizing the potential for high-quality science (Kirk, 2017). The 3Rs, first formulated by Russell and Burch (1959) in their book ‘The Principles of Humane Experimental Technique’ have become central to how the use of animals in scientific procedures is socially understood, politically imagined, and (inter)nationally regulated (Davies et al., 2018). They exist as a means of weaving together ‘good science, good care, and socially acceptable practices’ (Davies et al., 2018, p. 607), providing a pathway to aligning both ‘moral and scientific values within a pragmatic ethical framework’ (Kirk, 2017, p. 21).

At present, horseshoe crabs are outside of the scope of most formal legislation regulating animal use; not considered a ‘protected’ animal. Horseshoe crabs are a wild invertebrate, managed as a fishery, and bled through a process widely imagined as both non-invasive and a force for conservation, in order to produce a reagent that is readily positioned as an *in vitro* alternative in the first place (see also, Gorman, 2020). Hence, thinking about horseshoe crabs as a 3Rs issue has rarely been a priority to date.

Yet, there is growing interest amongst stakeholders in more fully engaging with a 3Rs framework (Bolden and Smith, 2017; Marius et al., 2020; Piehler et al., 2020). Bringing conversations about horseshoe crabs into connection with wider discussions about the 3Rs is seen as offering valuable opportunities for restructuring debates about biomedical horseshoe crab use, highlighting the efforts made within industry to improve practices. However, different stakeholders see different value and possibilities in each of the individual ‘Rs’, to the point of substantial friction between those who advocate focus on ‘replacement’ above ‘reduction.’ Ideas about replacement have tended to dominate discussions, a focus that has detracted from equally important efforts toward developing alternative approaches involving reduction and refinement.

As its central research question, this article queries what role the 3Rs – the ambition to replace, reduce, and refine the use of animals – might play in driving more sustainable methods within the biomedical utilization of horseshoe crabs? Additionally, the article considers what a social science perspective might add to discussions about the biomedical use of horseshoe crabs, and what might be learnt by bringing discussions about horseshoe crabs into conversation with the wider literature around the 3Rs? Thus, in what follows, the article moves to assemble stakeholder perspectives on opportunities for thinking with the 3Rs, considering current appetites for the development, promotion, and implementation of more sustainable methods for, and alternatives to, the use of horseshoe crabs for biomedical purposes.

## Social Scientific Methods for Investigating Perspectives on Alternatives and the 3Rs

Social science is, broadly, the study of human society and social relationships. Social scientists explore how people interact with one another, how shared cultures develop, and how people influence the world. Social science can help explain how society works, what different groups expect, and inform governance and policy. Social scientists working within the broad field of ‘science and technology studies’ seek to understand how scientific research and technological innovation are affected by, and affect in turn, society, politics, and culture. This involves exploring the different ways that scientific knowledge is produced and contested, and how different ‘claims’ to evidence, truth, and ethics are made and invoked in ways that influence practice (Cassidy, 2019). More recently, social scientists have become interested in how the use of animals in biomedicine is impacted by social, cultural, and political relations, and particularly, the contemporary challenges emerging as scientific practices and social expectations change (Davies et al., 2020). This involves examining how different interests are spoken for, and decisions made, around the biomedical use of animals.

The use of horseshoe crabs in endotoxin testing engages a complex range of stakeholder perspectives and involves multiple ‘epistemic communities’ each with their own shared beliefs, working practices, and criteria for assessing validity that lead them to form different understandings. Discussions about alternatives to horseshoe crab derived reagents involve considerable uncertainties and diverse views, with discussions becoming increasingly polarized (Guest, 2019). Krisfalusi-Gannon et al. (2018, p. 10) suggest that the drivers for horseshoe crab protection ‘are both environmental and economic.’ This may be true, but they are also social and cultural. As Davies et al. (2016) argue, social science research can make a significant difference to laboratory policy and practice, opening up understandings of the social, economic and cultural processes that influence practices surrounding the scientific use of animals, and the wider social contracts that enable public acceptance of the scientific use of animals. Social science can help understand the ‘shape of the conversation.’ This involves considering who is included in a conversation about the use of horseshoe crabs and how it is framed, focusing on understanding the various perspectives, positions, and sides of the debate in order to try and move discussions forward in productive ways (Cassidy, 2019). This is less about definitively identifying what is factually or morally ‘right’ and instead exploring what different stakeholders believe, and why they believe the things that they do (Cassidy, 2019).

In practice, this involved conducting qualitative interviews as a means of exploring perspectives on alternatives, sustainable methods, and the 3Rs. As Tiller et al. (2016, p. 4) suggest, when thinking about the management of marine resources, ‘there is a strong motive for engaging with stakeholders in order to access the expertise that they possess (i.e., "knowledgebase" data), which is characteristically strongly qualitative.’ Interviewing is one of the most commonly used qualitative research methods in the social sciences. In-depth interviews enable researchers to learn from interviewees’ perspectives, their situated and contextual experiences, and their attitudes and feelings toward – in this case – horseshoe crabs and endotoxin testing. Thirteen interviewees were selected from across the broad spectrum of groups with a stake in the biomedical use of horseshoe crabs: manufacturers, biotechnology companies, regulators, pharmaceutical scientists, conservationists, animal-welfare groups, academic researchers. This involved a level of purposive sampling – a commonly used sampling strategy in qualitative studies where respondents are selected to enable a subject to be studied in depth. These individuals were located across the United Kingdom, Europe, and North America. This approach enabled a narrow but deep focus. Interviews with these stakeholders explored their perspectives and concerns relating to the current and future roles of horseshoe crabs within practices of endotoxin testing. These interviews provide an opportunity to understand the priorities of stakeholders. Interviews lasted for an hour, on average, and were conducted via phone or online video-call. The research was assessed and approved by the University of Exeter’s Ethics Committee. The ‘semi-structured’ nature of the interviews allowed participants to focus on areas they felt were most important about their contextual – and uniquely situated – perspectives and concerns relating to the current and future roles of horseshoe crabs – and alternatives to crab-derived products – within practices of endotoxin testing. Semi-structured interviews involve a pre-prepared schedule of questions, however, they are also characterized by their flexibility, discursiveness, and open-ended nature, allowing the researcher to explore emergent ideas as the conversation progresses (Bryman, 2001). Some questions were asked to all interviewees, such as "How do you feel the landscape of endotoxin testing, and the use of LAL, has changed in recent years?" Whilst other questions were shaped dependent on the interviewee’s role regarding horseshoe crabs and endotoxin testing, with some specific to their sector (i.e., interviewees in the pharmaceutical sector were asked "What would motivate you to change to an alternative (non-animal) method of endotoxin detection").

All of these conversations were recorded, with interviewee’s consent, and transcribed to allow analysis. Participants received copies of their transcripts to review to enable accuracy and clarification. It is common practice within social scientific research to anonymize participants for reasons of confidentiality and ethical research practice. This is particularly the case here, given the sensitive nature of animals’ involvement in testing, along with the need for sensitivity around commercial interests. As such, all interviewees have been assigned attributions based on their broad sector of work. These have been broadly grouped and defined as the ‘biotechnology sector’ (*n* = 5) (stakeholders involved in producing endotoxin testing solutions, either LAL or alternative/recombinant methods)^2^, the ‘pharmaceutical sector’ (*n* = 3) (stakeholders involved in utilizing and conducting endotoxin testing as part of pharmaceutical production), the ‘conservation sector’ (*n* = 3) (stakeholders involved in advocacy, research, and practices aimed at conserving horseshoe crab populations), the ‘regulatory sector’ (*n* = 1) (stakeholders involved in setting and enforcing safety and standards), and the ‘communications and media’ sector (*n* = 1) (stakeholders involved in discussions about the use of horseshoe crabs from their perspectives as journalists and/or multimedia producers). However, it is particularly important to stress here that amidst these broad groupings, it is not possible to ascribe a singular view or sentiment to these categories – for example, as will be shown, whilst some within the pharmaceutical sector were positive about recombinant alternatives, others were still unconvinced.

Analysis followed a thematic analysis approach, utilizing NVIVO (a qualitative analysis software package). Thematic analysis is ‘a method for identifying, analyzing and reporting patterns (themes) within data’ (Braun and Clarke, 2006, p. 79). This involved comparing themes across different perspectives in order to understand what is important to different people, and identifying where there are differences and similarities between different groups. Through analyzing and examining different perspectives about evidence, validity, risk, we can begin to understand how different discourses about alternatives are forming, intersecting, and impacting practices.

## Perspectives on Reducing Biomedical Horseshoe Crab Use

“If you can replace 95% of your tests with a method that uses 99% less LAL, your impact is – I won’t go into the math – but it felt that it wasn’t unreasonable that a significant reduction could lead to a massive impact for the better.”

Interviewee, pharmaceutical sector

In biomedical research, reduction usually refers to ensuring that the minimum number of animals is used to answer the scientific question, using effective experimental design and statistical analysis to optimize numbers and avoid wasting animals. In the context of this use of the horseshoe crab, reduction can involve minimizing the number of animals that are required to be caught – or minimizing the amount of animal derived material used in a given method or process. Across stakeholder interviews, reduction was felt to be, as one interviewee from the pharmaceutical sector described, *’a big quick win, the sort of thing we’re looking at all the time.’* Reduction was perhaps the most palatable of the 3Rs, with the qualitative analysis showing that a majority of interviewees’ responses reflected a sentiment generally supportive of reduction (in principle), across the spectrum of stakeholders. Though the extent to which reduction could be achieved, how it could be achieved, and when it could be achieved, varied greatly. However, reduction is a framework which offers a progressive route for alleviating the burden placed on horseshoe crabs, but without requiring radical reconfiguration of existing practices. Reduction is increasingly framed as a process in contrast to replacement, for this exact reason, as Krisfalusi-Gannon et al. (2018, p. 9) argue, ‘revising the current system to improve efficiencies in horseshoe crab use may be more viable in the near term.’

The number of crabs utilized for biomedical purposes prior to 2004 are uncertain, since there was no standardized reporting, though the ASMFC estimate that medical usage increased from 130,000 crabs in 1989 to 260,000 in 1997 (2019, 35). Biomedical use has continued to grow, with the number of crabs delivered to biomedical facilities increasing from 335,501 crabs in 2004 to 575,760 crabs in 2017 (peaking with 622,098 in 2012). The ASMFC (2019, ii) suggests that biomedical use is now ‘fairly stable.’ However, there is concern that growing global demand (and emerging markets) for vaccines, pharmaceuticals and medical devices will require an increasing supply of LAL (Krisfalusi-Gannon et al., 2018). Whilst additionally, the rise of personalized medicine could necessitate individualized product testing on a per use basis, adding further pressure (Porzio, 2018). More recently, there is also concern that the COVID- 19 pandemic will increase the need for endotoxin testing, particularly in the face of vaccine production^3^.

The numbers of crabs collected for biomedical purposes are frequently contrasted against the earlier fertilizer industry – in 1880 over 4 million crabs were harvested from Delaware Bay alone (Kreamer and Michels, 2009), as well as the contemporary bait fishery, which utilizes horseshoe crabs as bait to catch eel and conch (whelk) – a process that involves a 100% mortality rate. Harvests here peaked in 1999 at 2.6 million horseshoe crabs, with 994,491 harvested in 2017) (Atlantic States Marine Fisheries Commission, 2019). This use of the animals was viewed as much more of a priority for replacement, reduction, and refinement, and 3 out of the 5 biotechnology respondents reported feeling aggrieved by what they described as ‘the vastly uneven attention’ placed on LAL manufacture, a view also shared by some pharmaceutical stakeholders:

“And the bait industry doesn’t get any media attention, that takes a million crabs and chops them up every year. It’s like can the bait industry reduce their reliance first?”

Interviewee, pharmaceutical sector

However, as Bolden (2019, p. 504) summarizes, it is ‘unproductive to pit the bait fishery take against the biomedical take.’ Indeed, as Davies (2019) has explored in the context of laboratory animals more broadly, drawing comparisons to the high numbers of animals used in other sectors does not reassure nor create a strong basis from which to argue an ethical point. However, interviewees felt that engaging with the specific numbers of crabs used biomedically did offer a route to creating a localized culture of care and interest in the 3Rs within endotoxin testing:

“When you translate it [reduction] back to crabs – very approximately because there’s so much variability – but when you can convert number of test vials and lysate for the crab, people are keen to hear that [...] we had challenges, and I said "just remember the drivers for change here, this is your forecasted burden reduction on the crabs and I understand this is difficult but if we can do this quicker, the impact is there" and that worked.”

Interviewee, pharmaceutical sector

Creating this engagement is important as the number of crabs bled is ultimately linked to demand and use, and thus the largest opportunities for reduction occur further down the supply-chain. There is huge scope – though presently, little awareness – for end-users in laboratories around the globe to effect reduction and significantly decrease the amount of crab blood used. AstraZeneca’s 2018 Sustainability report describes how as part of their ‘Culture of Care’ and focus on high standards of animal welfare they were keen to reduce the use of horseshoe crabs in testing, and have invested in new technology ‘which will see a reduction in annual lysate (crab blood reagent) consumption from approximately 7.5 litres to just a few hundred millilitres’ (AstraZeneca, 2018,48).

Technological fixes are regularly viewed as the way forward in terms of reduction. For example, one manufacturer of LAL has developed new technologies that allow the use of less raw material than traditional endotoxin testing methods. Charles River Laboratories argue that ‘if all tests were performed using cartridge technology, today’s entire worldwide LAL demand could be met with less blood than from Charles River’s current annual quota’ (Charles River Laboratories, 2020). Additionally, Guest (2019) advocates for the automation of endotoxin testing, suggesting it would result in a significant reduction in waste and in invalid tests that need repeating, along with the streamlining of testing plans to increase the number of tests per run, thus reducing total lysate used. Marketing for automation argues that ‘the most expensive LAL test is the one that must be repeated because of invalidity’ (Charles River Laboratories, 2020) – and this is also true in terms of the burden placed on crabs by testing errors.

There were also suggestions that optimizing the welfare of crabs might enable the collection of higher quality raw material, and thus offering pathways to reducing the number of crabs required to sustain the industry. This begins to blur the lines between reduction and refinement.

“The LAL that we’re getting in the wild probably isn’t the best LAL that could be available if the organism was fed appropriately and sufficiently, and managed in a temperature that was controlled, an environment that was controlled. So you can use less LAL, because – and it’s not even just an engineering function where you make the assay smaller – but because your source material is better. It can be higher in reactivity or activity and it can be better, so you can start to reduce the amount that you need and those things can start going toward the reduction component, if you’re making a better reagent in higher quantity, then that translates to needing less.”

Interviewee, biotechnology sector

This, however, is not straightforward, efforts to rear and maintain *Limulus* are wrought with welfare and economic challenges – as will be discussed more specifically in the later section on efforts for aquaculture-based refinements. However, even if a variety of these methods can be used to reduce the number of animals required, as an interviewee from the pharmaceutical sector reported, ultimately *"some people don’t believe that reduction’s enough, they want replacement, the ethical quandary of fishing these creatures doesn’t sit well with some people."* Yet perspectives on replacement are even more complex and contentious.

## Perspectives on Replacing Biomedical Horseshoe Crab Use

“Because of the 3Rs, there’s a move in the pharmaceutical industry to get animal sources of raw material, out of any raw material they use.”

Interviewee, biotechnology sector

Replacement refers to replacing animals with non-animal methods wherever feasible. A synthetic substitute to horseshoe crab blood was introduced in 2001 – laboratory-synthesized genetically engineered recombinant factor C (rFC) (Ding and Ho, 2001), becoming commercially available in 2003 (Bolden, 2019). Factor C here being ‘the endotoxin-sensitive serine protease that initiates the coagulation cascade’ in horseshoe crab blood (Ding and Ho, 2001, p. 277). However, initial uptake of this replacement was extremely limited due to the availability and market-dominance of the LAL test, combined with concerns about a single-source and supply of the synthetic, cautions over the validation of the alternative, and a lack of regulatory requirements to consider alternatives to testing in non-vertebrates.

More recently, there has been a renewed attention on replacements to the LAL test, emerging as a result of concerns relating to the sustainability of horseshoe crab populations and as recombinant reagents have become commercially available from multiple manufacturers (Bolden and Smith, 2017). One review of the performance of rFC as an endotoxin detection method suggested it is equivalent to, or better than, LAL in terms of the ability to detect and quantifiably measure bacterial endotoxin (Maloney et al., 2018). However, others have been less positive about the potential to move to this alternative on a routine or commercial basis, citing concerns about the current ability of the alternative to achieve adequate specificity (Dubczak, 2018). The topic of alternatives here has generated much discussion, with debates becoming increasingly polarized. Indeed, Guest (2019) (a scientist working on drug safety) describes inquiring about the technologies available for endotoxin testing, and receiving a ‘completely polarized message.’ Guest’s article goes on to explain, "in all honesty, with such a conflicting view, it was hard to trust the information" (Guest, 2019). This split was evident within stakeholder interviews too, with a divide between a belief that:

“Conversion to rFC would result in a 90% reduction in the demand for LAL, which means that mortality resulting from bleeding would decrease by an estimated 100,000 horseshoe crabs annually.”

Interviewee, conservation sector

And the contrasting idea that:

“Recombinant factor C is not going to “save” the horseshoe crab.”

Interviewee, biotechnology sector

Whilst scientific consensus over whether current replacement technologies are fit for purpose is still playing out, there is no question that their existence has changed the very shape of discourse around alternatives here:

“Before you couldn’t say that, now you can say it, particularly since there is an alternative, so it’s just changed the whole conversation. So now if the alternative is no good, that’s a different conversation, let’s talk about the efficacy of the alternative, but there **is** an alternative.”

Interviewee, conservation sector

Further, what we can see is the way in which the 3Rs framework is positioned as a driver for this move to seek a replacement. Bolden and Smith (2017, p. 405), note that ‘replacing the animal-derived LAL with rFC for endotoxin testing is consistent with the 3 R principles (replacement, reduction, refinement) for more ethical and sustainable use of animals for testing.’ They go on to explicitly link their interest in using recombinant factor C to replace LAL for endotoxin testing to their company’s ‘commitment to animal welfare and conservation.’ This link to animal welfare is particularly novel in terms of discussions of *Limulus*^4^. Though others questioned whether this turn to welfare and the 3Rs is an attempt to capture market-share through a cynical branding move:

“These companies have an alternative and they can market it to their marketing people as how it saves horseshoe crabs and all that, you wonder if they really, at the end of the day with all the marketing people aside, do they really believe it?”

Interviewee, conservation sector

One particular challenge here is that the shape of the conversation about replacement is focused on the idea of ‘saving’ the horseshoe crab – as can be seen in several quotes above. As such, discussions are mired in an unconstructive rhetoric that leads to defensive comparisons with other aspects affecting crab population vulnerability:

“So let’s not talk about the biomedical industry, let’s talk about erosion, let’s talk about development, let’s talk about all of these things in terms of protecting the horseshoe crab population. I’m willing to accept that the biomedical industry does have some horse in that race but I’m also convinced that they’re not the culprit here.”

Interviewee, regulatory sector

Whilst these are all valid concerns, and the horseshoe crab is indeed threatened by multiple compounding factors, this obscures arguments for replacement that are about reducing suffering to individual animals or improving animal welfare. Discussions about replacements for horseshoe crab blood would be better to frame themselves in terms of how they are restructuring the harm-benefit equations involved in the biomedical use of these animals (Davies, 2018).

There is also concern that a turn to synthetic alternatives might actually result in more harm to horseshoe crab populations; rather than being a high-value ‘catch and release’ asset within the biomedical economy, the rise of alternatives may shift the crab’s status as a commodity solely to that of fishing bait. For example, Charles River Laboratories, a manufacturer of LAL, suggest on their website that:

“Many companies think that moving away from LAL to reduce the use of the HSC will help them comply with 3R principles. This idea should be carefully evaluated, as moving to rFC could produce counterproductive effects by endangering the conservation efforts [...] Without the need for LAL in biomedical use, the legal protection ofthe horseshoe crab is not guaranteed in the future, and they would again fall prey to overfishing and use as bait.”

Charles River Laboratories, 2020.

Conservation is positioned here as a way of practicing care, performing stewardship, and offsetting harms to some crabs through providing affordances to the species at large. However, the idea that horseshoe crabs are only afforded protection and conservation by an ongoing exploitation of the species is one that did not appeal to everyone, and adds another level of complexity and contestation around the replaceability of horseshoe crabs. The likelihood of a rise in the bait fishery as a result of biomedical reduction or replacement is debated, given that there are already strict quotas on the bait industry.

In highly regulated areas, like the pharmaceutical industry, transforming the 3Rs from principles into practice can be a challenge (Törnqvist et al., 2014). In the case of rFC, regulatory approval has been slow to emerge. The FDA issued guidance allowing for the use of recombinant factor C instead of LAL- based assays in 2012, though this was still considered an ‘alternative test’ and subject to validation requirements, rather than considered a fully equable replacement (Levin, 2019). This was followed by revisions to the European Pharmacopoeia in 2016, which included recombinant factor C (rFC) as an alternative method, again subject to validation requirements. However, this amendment specifically attested that ‘the use of alternative reagents such as recombinant factor C as a replacement to the amebocyte lysate eliminates the use of a reagent extracted from live animals.’

However, there is concern amongst pharmaceutical scientists (66% of those interviewed) that the additional validation involved in using the replacement requires a considerable amount of additional time and expense. As one interviewee from the pharmaceutical sector explained, ‘*the likelihood of any company doing a validated alternative is not great because of the amount of validation that is required’* and that *’unless it was included in a pharmacopeial method’* they were unlikely to consider replacing their LAL use. Others argued that whilst the validation process was something of a hurdle, the extent of this had been greatly inflated, and could be negated over time through experience and a corporate commitment to animal welfare above what was easy.

“A lot of the other companies are out there marketing against it saying, “you’re going to have to spend all this money revalidating your methods,” and the reality is we can knock out a method validation in 2 days, instead of the 1 day that it takes. It’s four experiments instead of one, right? It’s not a huge amount.”

Interviewee, pharmaceutical sector

Whilst scientific and industry consensus over rFC is still playing out, in November 2019 the European Pharmacopoeia (Ph.Eur) Commission designated the ‘test for bacterial endotoxins using recombinant factor C (rFC)’ as a compendial method, effective as of July 2020 (Edqm, 2019). This will, at least within Europe, put the replacement test on an equal footing with crab-blood tests. However, pharmaceutical manufacturers operate in a globalized market, and without harmonization across the various Pharmacopoeias, there is still a long road for the alternative to gain industry confidence and uptake.

“So that specifically will help, if you’re a small European based manufacturer and you only sold in Europe. You could immediately switch to that and that would be great. Directionally, it’s great, it’s awesome and we’re very supportive. However, it’s tough for us because we operate globally.”

Interviewee, pharmaceutical sector

Concerns over patient safety were for many the bottom line. In a conservative, risk-averse sector, whilst many were encouraged by the promise of replacement, there was a desire for more data to emerge before people would feel confident to make this transition^5^.

“I can’t emphasize enough how much that patient centric approach is personally for me, it’s critical, I don’t want to have to question myself that I got it wrong. I’m sure it’s fine! But I’d like to see more data on it and I think there will be some more data coming out.”

Interviewee, pharmaceutical sector

Questions remain as to what level of evidence is required to achieve this confidence, and how to achieve industry acceptance. Some argued that much of the desired evidence is already available, and thus, the focus may need to be on education, improving access to existing evidence, and better communication of data.

“That’s where the effort needs to go and we think there’s an overwhelming amount of data that supports it, it’s just overcoming some of the political realities I think now, and just get in there [...] There’s actually a good deal of data out there and so we’re just trying to hope to direct people to that body of work, to show them there has been a lot of data out there and published.”

Interviewee, pharmaceutical sector

Ultimately, replacing LAL will take time, confidence, and data. For that reason, there are huge opportunities for refinement to the process, as one interviewee from the conservation sector concluded, *’as we are going to continue to bleed animals, as we make this migrationfrom LAL to rFC, we should do it as humanely as possible.’*

## Perspectives on Refining Biomedical Horseshoe Crab Use

“What they’ve been looking at is a way of finding a less invasive way of taking blood from the animal and also they wanted to look at the stressors that the animal goes under through the entire process, from being harvested to being bled to being released, I know there’s been a lot of papers done on that but nobody’s really gotten into the changes that are going on within the animal itself.”

Interviewee, conservation sector

Refinement involves reducing suffering and improving welfare throughout animals’ lives, including during and after procedures, and within transport, housing, handling, husbandry and care. Because of the multiple stages involved in procuring horseshoe crab blood – collection, transportation, storage, bleeding, re-release – there is considerable scope for refinement of processes. Krisfalusi-Gannon et al. (2018, p. 5) note that the percentage of horseshoe crabs that died prior to being bled more than doubled between 2008 to 2012, and suggest that a culture of deleterious harvest and transportation practices exists. Recently, Owings et al. (2020, p. 225) have argued that ‘if biomedical facilities take sex differences and seasonal fluctuations in HCY (hemocyanin) levels into account, they might be able to reduce some of the deleterious effects of the bleeding process.’

Much of the work refining processes regarding the biomedical use of horseshoe crabs has gone quietly unnoticed, thanks to the industry’s tendency toward secrecy. However, the ASMFC’s ‘best management practices’ introduced in 2011 represent a significant step-change in how the welfare of individual crabs was considered at each stage within the collection, bleeding, and release of crabs collected for biomedical purposes.

“I go out with our fishermen and I audit their practices. In our contract with them, we have it specified as per the best practices document and so it’s actually in our contracts with our fishermen on how they are to handle the horseshoe crabs. They’re treated very gently and they’re brought back to the same spot where they were taken, within 24 h, the shells are marked so they’re not re-bled in the same year.”

Interviewee, biotechnology sector

Whilst some were reassured by the stated efforts toward refinement, and the acts of care they involved, this was not shared by everyone. As one interviewee from the conservation sector remarked, *’you can find the guidelines and stuff, the recommendations, the best practices but that’s not to say that they’re actually following those.’* Several manufacturers commented that they are routinely audited and inspected by regulators, with strict mandates and conditions of operation imposed at State levels. However, at a broader (public) level, opportunities for witnessing refinement are limited, and with little openness in the sector, much has to be taken on good faith that moves toward more refined, less harmful, methods are taking place. Future work might involve an independent and public assessment of the extent of the implementation of these best management practices. Indeed, at present, there is a hesitancy to explore refinement in case this implied an admittance or acceptance that current standards and practices were not adequate at safeguarding animal welfare.

“That’s a hard thing to get them to swallow, to change their operational position and that would further have to make them kind of suggest that their processes, to some extent, are deleterious to the species. And can they say that?”

Interviewee, biotechnology sector

Krisfalusi-Gannon et al. (2018, p. 4) suggest that ‘improved harvest practices have the potential to reduce mortality rates during biomedical harvest by more than half.’ Krisfalusi-Gannon et al.’s (2018) paper is a highly novel investigation into opportunities for implementing sustainability and welfare considerations into the supply chains of *Limulus* blood. They question a range of possibilities from removing a smaller volume of blood per drawing, to the scope for using indwelling catheters, and even the potential to develop processes of plasmapheresis and reinfusing crabs. However, it is still early days for refinement in this area. Krisfalusi-Gannon et al. (2018, p. 9) go on to argue that ‘to achieve the lowest horseshoe crab mortality and highest blood quality during biomedical bleeding, a more systematic understanding of the nuances of the optimal horseshoe crab environment, feeding and care would be required.’

Other movements toward refinement in this area have involved exploring the potentials of aquaculture and the maintenance of captive populations. There are relatively few published studies that discuss husbandry conditions for horseshoe crabs, and many researchers consider ‘captive rearing to be difficult, time consuming and impractical’ (Carmichael and Brush, 2012, p. 39). To date, most conceptualizations of horseshoe crab aquaculture have been framed as stock enhancement through the release of juveniles into the wild (Schreibman and Zarnoch, 2009). Crabs are slow to reach maturity, and many studies report chronic mortality after 6 months in captivity (thought to be linked to diet) (Carmichael and Brush, 2012). Aside from the obvious implications on animal welfare, these challenges also pose significant economic barriers. Despite these challenges, there are aspirations that creating what might be understood as a laboratory ‘strain’ of horseshoe crab, as opposed to the current wild usage, might offer opportunities for greater care and welfare.

“We started to aquaculture horseshoe crabs, provide them an optimized management and optimized feed, with the hopes of going a low impact resource harvesting and we have some interesting ideas, that fall in probably the first or second R, it’s not necessarily in that Replacement R.[...] That’s the luxury we have here because after [bleeding], we then have the opportunity to be three steps from them and care for them and then feed them, whereas opposed to just saying ‘you’re on your own now’.”

Interviewee, biotechnology sector

Aquaculturing might offer opportunities to remove the stresses induced by capture and transport of wild stock. Similarly, Tinker-Kulberg et al. (2020) suggest that aquaculture would allow for taking smaller amounts of blood, more frequently, rather than the current system that can involve collecting ‘between 25 and 40% of a crab’s blood (Moore, 2017, p. 112). The effect that the amount of blood extracted has on mortality rates, and what the upper limits of this should be for ‘best practice,’ remains a contested area (Atlantic States Marine Fisheries Commission, 2019). Whilst taking less more frequently, may be appealing at first, there are also ethical concerns about the cumulative effect on potential animal suffering, something which must be considered in any harm-benefit analysis of ‘refinements’ (Davies, 2018). Despite this, Tinker-Kulberg et al.’s (2020, p. 9) experiments found that aquaculture approaches seemed to indicate ‘healthier and less stressed animals,’ resulting from an optimized environment. Rather than a brief and extractive relationship, aquaculturing crabs might allow for more caring relationships with crabs. One that is less ‘parasitic,’ as Moore (2017) suggests of contemporary practices. Of course, other ethical arguments might question what freedoms are being lost through an enforced captivity.

Some expressed a concern too that large-scale efforts at refinement, like aquaculture, detracted from smaller, quieter, efforts that might improve horseshoe crab welfare, efforts such as training or auditing, that might contribute more to the establishment of a culture of care for crabs. Ultimately however, whilst there are burgeoning efforts toward refinement, stakeholders were also downcast about the possibilities of these refinements being taken up within industry at large, particularly from the perspective of the additional costs associated with aquaculture compared to wild-catch:

“I don’t necessarily think that we can change the way that the biomedical industry is harvesting and I think that that’s a tall order, when they’re paying fishermen a little bit of money to go collect them and put them in the vehicles and bring them back, it’s basically a free resource for them so if we were to go in and say, "invest money and do all of these things, it will improve your image and it will safeguard the future of your business." I personally don’t necessarily think that it’s a feasible task to get them to change their mind but maybe we can get the people that buy it to change their mind on where they buy from, to where the animal is at least treated ethically throughout their donation process.”

Interviewee, biotechnology sector

As the above quote draws attention to, it is easy to place all of the burden of welfare on the manufactures of LAL who bleed the crabs, however, everyone within the pharmaceutical supply chain who uses LAL is implicated in contributing to horseshoe crab welfare. This may involve developing communication strategies that highlight that LAL is derived from a living animal, taking steps to ensure efficient and non-wasteful use, exploring opportunities to replace, reduce, or refine use, and questioning and holding suppliers to account about how welfare considerations are implemented in their manufacture of LAL.

## Discussion

Creating more ethical and sustainable futures for humans and horseshoe crabs alike will require changing the shape of conversations about horseshoe crabs. The use of existing ethical, regulatory, and conceptual frameworks like the 3Rs offers huge potential to reframe discussions and find ways to talk about the biomedical use of horseshoe crabs that avoid the growing polarization, whilst introducing a means of extending – and conveying – the welfare considerations that are increasingly expected across science and society. Importantly, this should be viewed as the 3Rs in concert, as one interviewee from the biotechnology sector concluded:

“I like your 3Rs because I’m involved in all of them. They’re all important and the thing is that everybody has to recognize that all of them are important and they all interact.”

Interviewee, biotechnology sector

As Prior et al. (2019, p. 11) conclude, ‘it is clear that there are still many opportunities to replace, refine or reduce the use of animals in pharmaceutical safety evaluation.’ Endotoxin testing, and its reliance on horseshoe crabs is no exception. There are exciting efforts emerging to replace, refine, and reduce contemporary biomedical reliance on horseshoe crabs. The extent to which a turn to welfare and the 3Rs exists as an attempt to capture market-share may be debated. Certainly, the choice of ‘R’ here is often heavily influenced by economic interests, with existing manufacturers keen to innovate technological fixes that move toward some semblance of reduction, but ultimately, maintain the *status quo* of crab-bleeding, whilst the replacement products serve to create a pathway for new players to enter a market reportedly valued at $462.38 million in 2014 (Moore, 2017, p. 119). However, it remains the case that efforts to innovate here do offer the potential for improving the level of humane care deployed in the biomedical utilization ofhorseshoe crabs – something that is increasingly expected by publics. These expectations of ‘good’ care will remain on the agenda as the knowledge of the use of these ancient animals grows within public understandings of, and engagements with, science.

Luo et al. (2020, p. 11) suggest that ‘we need additional cooperation to discuss and improve horseshoe crab research.’ This cooperation should increasingly be interdisciplinary. This research has demonstrated the value that a social science perspective can bring to understanding perceptions about the development, promotion, and implementation of more sustainable methods for, and alternatives to, the use of horseshoe crabs for biomedical purposes. Whilst this research has taken a narrow and deeply focused approach, working with key stakeholders, to understand the ‘anatomy of arguments’ around horseshoe crab use, there is great potential for future work to incorporate a mixed methods approach, including quantitative analysis of responses to map and poll attitudes more widely. By unpacking what the meaningful questions may be to ask, this research sets the stage for future, more quantitative, work. Further work to include a focus more inclusive of the TAL sector too would help. Better understanding how different stakeholders perceive, and make value judgments about, horseshoe crabs – as strange, distant, invertebrates – is necessary to create more sustainable futures. The use of horseshoe crabs in endotoxin testing is a complex scientific and societal issue, situated at the interface of human, animal, and environmental health. Addressing emerging questions in global public health, which intersect with ecological concerns and ethical issues, requires novel interdisciplinary collaborations involving social science.

As discussed earlier, although scientific consensus over whether current replacement technologies are fit for purpose is still playing out, there is no question that their existence has changed the very shape ofdiscourse around alternatives here:

“I think what’s been really good for the industry over the past few years is that the discussion is there on the table, which it wasn’t prior to 2016, everyone was just sort of taking it for granted. There was a bit but it was just people went merrily on their way so I think it’s good that we’ve got the discussion on the table.”

Interviewee, pharmaceutical sector

It highlights how conversations about sustainability, care, welfare, and replacing, reducing, and refining the current use of horseshoe crabs are here to stay. Requests for more data about the efficacy of recombinant factor C, along with a desire to await the development of more complex alternatives that involve recombinant formulations of the other factors involved in the clotting cascade within ‘natural’ horseshoe crab blood (such as Factor B and pro-clotting enzymes) will no doubt continue to shape discussions. However, this demonstrates the direction that the industry is moving – ultimately, toward more sustainable methods. Questions are increasingly less about could, or should, horseshoe crab blood be replaced, but more about when, and what the threshold of confidence, data, and trust, might be to do this. This discursive move is a significant achievement for all of those concerned about the animal welfare and environmental sustainability impacts of current LAL testing.

There is still a long road for alternatives and replacements to gain industry confidence and uptake, but being a ‘compendial test’ in Europe represents a significant milestone in the use of non-animal methods. The European decision is a positive result for a marine species afforded little protection or welfare considerations, despite – as social media reactions to articles about horseshoe crab use regularly demonstrate – a public desire to see more care expressed in the biomedical use of animals. Importantly, this social expectation of care is not just for those animals we find deeply familiar or appealing, but also for enigmatic invertebrates like horseshoe crabs.

## Data Availability

The datasets generated during the study are being prepared for deposit to the UK Data Archive at the end of the project and are not currently publicly available. Anonymized interview transcripts from participants who consented to data sharing are available from the corresponding author, subject to reasonable request. Requests to access the datasets should be directed to “r.gorman@exeter.ac.uk.”

## Footnotes

1 Global endotoxin detection is also dependent upon the Tachypleus amebocyte lysate (TAL) test, produced in China, and utilized for analogous testing by Asian and Pacific-based pharmaceutical manufacturers (Krisfalusi-Gannon et al., 2018). However, this involves a complex geography, as many multi-national pharmaceutical companies that manufacture products in China ultimately test their end products with LAL as they are selling worldwide (Gauvry, 2015). This involves the utilization of *Tachypleus tridentatus* (commonly known as the Chinese horseshoe crab, Japanese horseshoe crab, or tri-spine horseshoe crab), a species which is listed as endangered (Laurie et al., 2018). With the population of *T. tridentatus* declining drastically there is speculation that this could lead to a compensatory spike in the global demand for LAL (Krisfalusi-Gannon et al., 2018). There is concern that ‘regulations to protect the harvest of crabs for biomedicine are not as successful in Asia as in the United States,’ and that bleeding practices often involve ‘draining to death,’ rather than the catch and release fisheries practiced in America (Moore, 2017, p. 120; Laurie et al., 2018). Thus, although this article focusses specifically on the Atlantic horseshoe crab (*Limulus polyphemus*) and the Limulus Amebocyte Lysate (LAL) test, it will be increasingly prudent to look at the 3Rs and issues of animal welfare across all species of horseshoe crabs and forms of endotoxin detection solutions.

2 Some biotechnology companies have a portfolio that includes manufacturing both LAL and rFC endotoxin testing solutions.

3 Whilst this has been widely speculated, in interviews with Arnold (2020) in *National Geographic,* one manufacturer is quoted as explaining "that to make five billion doses of the COVID-19 vaccine, 600,000 tests will be performed, which will use the amount of lysate created in a single day," something they go on to argue would place no undue burden on either the lysate supply chain or horseshoe crab populations. Regardless, the pandemic has brought renewed attention to the many animals that are entangled in ensuring human health.

4 Whilst some would argue that from the outset ‘LAL manufacturers were concerned about the welfare of the horseshoe crab’ (Novitsky, 2002, p. 79), this often appears conflated with ideas of conservation. In such that the focus is themed around preservation, survival, and preventing *’Limuluspolyphemus* from going the way of the passenger pigeon and the dodo bird (Galler, 1979, p. 108), rather than welfare as a concept focused on the quality of life of animals (Fraser et al., 1997).

5 Some interviewees drew parallels to the original transition from the Rabbit Pyrogens Test to LAL: “when you look at it, it was probably a good 15 or more years before the LAL test was really accepted as a substitute for the rabbit pyrogen test.”
